# Kindergarten teachers’ compassion fatigue: decreasing with empathic ability or empathic motivation or both?

**DOI:** 10.3389/fpsyg.2025.1717699

**Published:** 2026-02-05

**Authors:** Fangyan Chen, Wenjun Xu, Yabo Ge, Weijian Li

**Affiliations:** 1 Zhejiang Normal University, Jinhua, China; 2 Jinhua University of Vocational Technology, Jinhua, China

**Keywords:** compassion fatigue, empathic ability, empathic motivation, empathy deficit, kindergarten teachers

## Abstract

Compassion fatigue, often referred to as the “cost of caring,” reflects a form of empathy deficit. However, it remains unclear whether it stems from a diminishment in empathic ability, empathic motivation, or both. To address this gap, we developed a hierarchical regression and structural equation model using survey data from 1,161 kindergarten teachers. The findings revealed that both empathic ability and empathic motivation significantly and negatively predicted compassion fatigue. Specifically, job burnout was negatively predicted by both empathic ability and empathic motivation, whereas secondary stress was negatively predicted by empathic motivation but not by empathic ability. Theoretical and practical implications, along with directions for future research, are discussed.

## 1 Introduction

Witnessing others’ suffering can prompt individuals to empathize through acts of caregiving, concern, or compassion (Batson, 2010). Yet the act of sharing another’s pain, while beneficial to the sufferer, may also pose risks for the observer, leading to personal distress or even compassion fatigue (Decety et al., 2010; Figley, 1995). As a condition that undermines the psychological well-being of those who empathize, compassion fatigue has garnered substantial scholarly attention in recent years (Hansen et al., 2018; Ormiston et al., 2022; Roy and Naik, 2025; Vaccaro et al., 2021).

Nevertheless, the phenomenological understanding of compassion fatigue remains debated. Some scholars conceptualize it primarily as a reduction in empathic ability. For instance, Nolte et al. (2017) described compassion fatigue as a form of exhaustion that undermines one’s capacity to maintain caring relationships. Peters (2018) likewise viewed it as a decline in empathic ability resulting from repeated exposure to others’ suffering, and Ormiston et al. (2022) similarly defined it as diminished empathic capacity following exposure to others’ experiences. Other researchers, however, have emphasized the importance of empathic motivation. For example, Chen et al. (2023) highlighted the central role of empathic motivation in the development of compassion fatigue, and Nelson et al. (2003) argued that compassion fatigue weakens empathy not by impairing ability but by reducing individuals’ motivation to invest the necessary cognitive effort. Still, Figley (1995) characterized compassion fatigue as a decreased ability or willingness to be empathic after encountering others’ traumatic events. Along similar lines, Adams et al. (2006) defined it as a caregiver’s inability or lack of desire to empathize or share in clients’ suffering, and Sun et al. (2011) described it as reduced interest and ability to empathize with those who are suffering. Taken together, these perspectives underscore an unresolved question: with respect to empathy, does compassion fatigue reflect a decline in empathic ability, a loss of empathic motivation, or a combination of both?

### 1.1 Compassion fatigue as an empathy deficit

Although its definition remains contested, compassion fatigue is commonly framed as a deficit in empathy—manifested as depersonalization or dehumanization arising from the distress of helping, caring for, or empathizing with others (Vaccaro et al., 2021). A wide range of professionals, such as nurses (Arnold et al., 2025; Ledoux, 2015), healthcare workers (Bhugra and Ventriglio, 2024; Okon et al., 2025), counselors (Nas, 2025), and even teachers (Kendrick et al., 2025; Sayers and Anderson, 2025), has reported experiencing compassion fatigue in response to sustained emotional demands. Compassion fatigue is often accompanied by physical symptoms (e.g., sleep difficulties, headaches), behavioral reactions (e.g., avoidance, increased alcohol use), and psychological consequences (e.g., depression, emotional exhaustion) (Beck, 2011; Bhugra and Ventriglio, 2024; Figley, 2002; Sinclair et al., 2017).

How do social and emotional workers come to develop compassion fatigue? According to the seminal model of compassion fatigue proposed by Figley (1995), the development of compassion fatigue begins with consistent empathy. These caregivers, accustomed to exposure to others’ suffering, must have the capacity for empathy and feel motivated to alleviate it. However, each empathic response depletes their reserves of empathic energy, such as cognitive and emotional cost (Cameron et al., 2019; Spaulding, 2023). Thus, empathy and the stress it generates interact with other negative influences to produce compassion fatigue (Figley, 2002). Thus, while empathizing with others is a precondition for compassion fatigue, it’s typically characterized as an empathy deficit (Chen et al., 2025).

### 1.2 Empathic ability and empathic motivation in understanding empathy deficit

A range of definitions and manifestations suggest that compassion fatigue can be understood as a form of empathy deficit (Bhugra and Ventriglio, 2024; Ormiston et al., 2022; Sun et al., 2016). Traditional explanations have largely attributed such deficits to insufficient empathic ability (Seppälä et al., 2017; Weisz et al., 2021). Yet, growing scholarship challenges this narrow view by emphasizing another essential component, empathic motivation (Ferguson et al., 2021; Ge et al., 2023; Schumann et al., 2014; Weisz et al., 2022; Zaki, 2014). For example, Zaki (2014) proposed an empathic motivational framework, noting that empathy is a motivated phenomenon for which at least three approach motives (positive affect, affiliation, and social desirability) and three avoidance motives (suffering, material costs, and competition) drive people to approach or avoid empathy.

Similarly, the empathic propensity–ability dissociating theory, a seminal theory proposed by Keysers and Gazzola (2014), divides empathy into two components: ability and propensity for empathy. They contend that empathy emerges through the interaction of these two components and that deficits may arise when either or both are absent (Weisz and Cikara, 2021). Numerous empirical studies in recent years have provided support for this theoretical perspective. For example, children with advanced theory-of-mind skills may deploy these abilities manipulatively rather than empathically, illustrating that ability without motivation does not necessarily lead to empathy (Doenyas, 2017). Similar patterns appear in individuals with dark personality traits, who generally understand others’ emotions but have little inclination to empathize (Kajonius and Björkman, 2020). Furthermore, Cameron et al. (2019) posit that people seek to avoid empathy because of its cognitive costs, and they refer to this phenomenon as the motivational effect on empathic choices. However, this effect could be offset by increasing empathic benefits (Ferguson et al., 2020). Broadly, this body of theoretical and empirical work establishes that empathy is a motivated phenomenon (Zaki, 2014), and whose activation involves an empathic cost–benefit trade-off (Ge et al., 2023). Consequently, both empathic motivation and ability are posited as critical determinants of empathy deficit (Ferguson et al., 2021; Keysers and Gazzola, 2014; Tremblay et al., 2021; Weisz and Zaki, 2018; Zaki, 2014).

### 1.3 Kindergarten teachers are particularly susceptible to compassion fatigue

Compassion fatigue has been widely documented across a range of helping professions, including nurses (Çakmak et al., 2025; Potter et al., 2010), physicians (Fernando and Consedine, 2014), and even educators (Kendrick et al., 2025; Koenig et al., 2018). Teaching, in particular, is a profession characterized by intensive interpersonal interaction and sustained emotional labor, requiring teachers to provide ongoing social and emotional support to students on a daily basis (Jennings, 2011; de Ruiter et al., 2021; Wieck et al., 2021). As a result, teachers are also highly vulnerable to compassion fatigue (Lombard et al., 2025; Sayers and Anderson, 2025; Xue et al., 2025), yet their experiences are often overshadowed by the extensive research attention given to healthcare workers. This vulnerability is even more pronounced among kindergarten teachers, who routinely encounter young children’s distress, suffering, and emotional volatility—experiences that demand continuous empathic engagement (Fan et al., 2016). Consequently, kindergarten teachers exhibit elevated levels of stress and report both psychological and physical strain (Herman et al., 2018). For example, Li et al. (2020) found that more than half of Chinese kindergarten teachers experienced burnout due to chronic and sustained occupational pressure. The high frequency and emotional intensity of child-related empathic situations place kindergarten teachers at substantial risk for compassion fatigue (Cai et al., 2025; Ma et al., 2021; Qi et al., 2015; de Ruiter et al., 2021).

Meanwhile, these challenges are exacerbated by broader demographic and policy developments. In line with the National Plan for Medium- and Long-Term Education Reform and Development extending to 2035, China has significantly expanded kindergarten teacher recruitment. The number of kindergarten teachers rose from 2.43 million in 2017 to 2.83 million in 2024. However, the population of age-eligible children (3–6 years old) increased even more sharply during the same period, from 25.49 million to 35.84 million (Ministry of Education in People’s Republic of China, 2025). Such high child-teacher ratios expose kindergarten teachers to greater levels of distress and adverse emotional experiences, as young children require consistent empathic engagement on a daily basis. Taken together, these factors converge to place kindergarten teachers among the professional groups most susceptible to compassion fatigue (Cai et al., 2025; Chen et al., 2023; Chen et al., 2025).

According to the two-factor model of compassion fatigue proposed by Figley (2002), compassion fatigue comprises secondary stress and burnout. Burnout refers to a state of prolonged physical, emotional, and mental exhaustion, often accompanied by hopelessness, reduced professional efficacy, and increased cynicism (Maslach and Leiter, 2016). In contrast, secondary stress involves symptoms such as heightened fear, intrusive recollections, and avoidance behaviors (Figley, 2002). Aligned with this model, substantial research has established that both components significantly undermine teachers’ emotional regulation and well-being (Oberg et al., 2025; Zhou et al., 2025). For instance, Gecewicz et al. (2025) found that higher burnout levels were significantly associated with stronger intentions to leave the profession. Meanwhile, Hydon et al. (2015) demonstrated that secondary stress can impair teachers’ mental health, emotional stability, and interpersonal relationships. Despite both being detrimental, some differences exist between them. For example, burnout is a stronger predictor of turnover intent, whereas secondary traumatic stress, despite its impact on well-being, does not reliably predict leaving the profession (Christian-Brandt et al., 2020). Consequently, to comprehensively understand the relationship between compassion fatigue, empathic ability, and motivation among kindergarten teachers, it is essential to analyze secondary stress and burnout as distinct components.

### 1.4 The present study: aims and hypotheses

The present study seeks to clarify the contributions of empathic ability and empathic motivation to compassion fatigue, thereby addressing an ongoing debate regarding whether compassion fatigue is influenced primarily by one of these components or by both. Additionally, it aims to examine how the two subcomponents of compassion fatigue, namely, secondary traumatic stress and burnout, relate to empathic ability and empathic motivation. To investigate those questions, we focused on kindergarten teachers, who are a population particularly vulnerable to compassion fatigue, to examine how these two facets of empathy relate to their experience of it. Using a cross-sectional design, we assessed participants’ empathic ability, empathic motivation, compassion fatigue, and demographic information, and employed structural equation modeling to test the proposed relationships.

Based on Figley’s model of compassion fatigue (Figley, 1995) and several previously mentioned theories on empathy (Cameron et al., 2019; Ge et al., 2023; Keysers and Gazzola, 2014; Zaki, 2014), we hypothesized that both empathic ability and empathic motivation would significantly predict compassion fatigue. By formally evaluating this hypothesis, the study aims to advance understanding of the mechanisms underlying compassion fatigue and to offer empirical evidence that can inform the development of theory-based interventions to support teachers’ psychological well-being. Ultimately, we expect that the findings will provide actionable insights for designing practical strategies to mitigate compassion fatigue among teachers.

## 2 Method

### 2.1 Participants and procedure

The research procedures were conducted following the Declaration of Helsinki and were approved by the local Kindergarten Review board and Zhejiang Normal University Review Board (ZSRT2024093). This study employed a cross-sectional design, and the survey was administered online via the SOJUMP platform.^1^ Before completing the survey, all participants signed written informed consent forms and were guided on how to complete the survey, with their anonymity assured. Then, participants completed a demographic questionnaire, carefully completed the full scales, and were free to quit at any time.

We recruited 1,193 kindergarten teachers from Zhejiang province. The inclusion criteria comprised (a) at least 1 year of teaching experience and (b) current frontline teaching duties. Exclusion criteria included individuals holding primarily administrative positions, temporary or substitute teachers, and student teachers undertaking internships. For data quality control, 32 participants were excluded from the data screening process because of missing data for important study variables or completion times shorter than two standard deviations (*SD*) from the mean value. The final valid sample consisted of 1,161 kindergarten teachers, representing a 97.32% valid response rate. Participants had a mean age of 30.19 years (*SD* = 8.12), with a range of 21 to 58 years. The sample was predominantly female (98.6%, *n* = 1,145), with the majority working in urban areas (59.7%, *n* = 693). Regarding professional titles, 41.1% (*n* = 477) held no title, 34.7% (*n* = 403) held secondary titles, 22.5% (*n* = 261) held primary titles, 1.6% (*n* = 19) held senior titles, and one participant (0.1%) held a special title. The average teaching experience was 8.93 years (*SD* = 7.85), ranging from 1 to 40 years.

### 2.2 Instruments

Compassion fatigue for kindergarten teachers was measured using the Compassion Fatigue short scale (CF short scale) (Adams et al., 2006), which has been revised and normalized by Sun et al. (2014) and is extensively used in Chinese culture (Sun et al., 2016). This instrument, with a two-dimensional structure (i.e., job burnout and secondary stress), included 13 items that participants rated on a 10-point Likert scale (from “1 = *rarely/never*” to “10 = *very often*”). Higher scores indicate a higher intensity of compassion fatigue. To construct a more accurate assessment of compassion fatigue for kindergarten teachers, we have changed the statements of the targets. For example, we revised “I have felt a sense of hopelessness associated with working with clients/patients” to “I have felt a sense of hopelessness associated with working with students.” To validate the two-factor model, we conducted confirmatory factor analyses, which showed good structural validity (*χ*^2^/*df* = 14.19, RMSEA = 0.10, CFI = 0.89, TLI = 0.87, SRMR = 0.07). The factor loadings are detailed in the supplemental material. Further, the internal consistency was 0.90, and the two subscales in this study were *α* = 0.88 and 0.85, respectively.

Motivation for teacher empathy (MTE), developed by Ge et al. (2021), is a short questionnaire to assess the intensity of MTE in an educational situation in three ways (i.e., cognition, emotion, and behavior). This scale has three items (i.e., “when a child is in a bad mood, I want to know what they are thinking at that moment”) and is scored on a five-point scale, where 1 = “*strongly disagree*” and 5 = “*strongly agree*.” Higher scores were indicative of stronger MTE intensity. In this study, the internal consistency reliability coefficient was set at 0.75.

Ability for teacher empathy was measured using Perspective-taking (PT), a subscale of the Interpersonal Reactivity Index (IRI) (Davis, 1983). This scale is widely used in Chinese culture (Chen et al., 2018; Sun et al., 2018). PT comprises seven items (e.g., “I believe that there are two sides to every question and try to look at them both”). Participants rated their agreement or disagreement on a seven-point scale (1 = “*does not describe me well*,” 7 = “*describes me very well*”). In line with previous studies (Engen and Singer, 2013; Li et al., 2020), higher scores on the PT scale were associated with stronger teacher empathic ability. In this study, Cronbach’s alpha for this scale was 0.82.

### 2.3 Data analysis

Descriptive statistics and partial correlation analyses (i.e., to control for gender, age, teaching experience, and title covariates) were conducted to examine the associations among crucial variables using SPSS (version 23.0). Furthermore, a two-step hierarchical regression analysis was conducted to explore the role of empathic ability and motivation in compassion fatigue among kindergarten teachers. Specifically, demographic variables, including region, gender, age, teaching experience, and professional title, were entered in the first block. In the second block, empathic ability and empathic motivation were added to examine their incremental predictive effects on compassion fatigue above and beyond the demographic factors. This analytic strategy allowed us to determine whether empathy-related variables explained additional variance in compassion fatigue after controlling for basic demographic characteristics. Finally, to further test the role of empathic ability and motivation on the subscales of compassion fatigue, a structural equation model (SEM) was implemented using *Mplus* (version 7.0). In this model, job burnout and secondary stress were the dependent variables, while empathic ability and motivation were applied as predictor variables. The covariates were the same as those in the partial correlation analyses. Before conducting the hierarchical regression and structural equation modeling (SEM), preliminary assumption checks were performed. For normality, the absolute values of skewness ranged from 0.01 to 0.90, and kurtosis ranged from 0.15 to 0.91, which fell within the commonly accepted range for approximate normality. The Q-Q plots further indicated that the distributions of all core variables closely followed the reference line, supporting the assumption of normality. Regarding multicollinearity, variance inflation factors (VIFs) were examined for all predictors. The VIF values for Empathic ability, Empathic motivation, Job burnout, and Secondary Stress were 1.22, 1.19, 1.66, and 1.57, respectively, which indicated that there was no problem of collinearity between the variables (Thompson et al., 2017). Overall, the dataset met the assumptions required for conducting hierarchical regression and SEM.

## 3 Results

### 3.1 Preliminary analyses

As shown in Table 1, compassion fatigue was significantly negatively correlated with empathic ability(*r* = −0.18, *p* < 0.001) and motivation(*r* = −0.17, *p* < 0.001), and job burnout was negatively correlated with empathic ability (*r* = −0.22, *p* < 0.001) and motivation (*r* = −0.19, *p* < 0.001). Furthermore, secondary stress was negatively correlated with empathic motivation (*r* = −0.09, *p* < 0.01), but not empathic ability (*r* = −0.06, *p* = 0.054). Finally, the empathic ability was positively correlated with empathic motivation (*r* = 0.39, *p* < 0.001).

**Table 1:** Means, standard deviations, and correlation coefficient of variables.

Variables	M ± SD	1	2	3	4
1 Empathic ability	27.68 ± 4.48	—			
2 Empathic motivation	18.18 ± 2.71	0.39^***^	—		
3 Compassion fatigue	46.40 ± 21.74	−0.18^***^	−0.17^***^	—	
4 Job burnout	30.85 ± 14.66	−0.22^***^	−0.19^***^	0.94^***^	—
5 Secondary stress	15.55 ± 9.53	−0.06	−0.09^**^	0.84^***^	0.60^***^

^**^p < 0.01, ^***^p < 0.001.

### 3.2 Hierarchical regression analysis

To test our hypotheses, we conducted a hierarchical regression analysis with compassion fatigue as the dependent variable, as shown in Table 2. The regression coefficients are reported in their unstandardized form. We then entered covariate variables such as gender, age, teaching experience, and title as the first step in the model, followed by the predicted variables (i.e., empathic ability and empathic motivation) as the second step. The final models showed slight evidence that empathic ability (*β* = −0.60, *SE* = 0.15, *p* < 0.001, 95% *CI* [−0.90, −0.30]) and empathic motivation (*β* = −0.90, *SE* = 0.25, *p* < 0.001, 95% *CI* [−1.40, −0.41]) were significant predictors of compassion fatigue.

**Table 2:** Hierarchical regression analysis among the key variables.

Variables	First step			Second step		
	*β*	SE	95% CI	*β*	SE	95% CI
Areas	1.84	1.30	[−0.72, 4.40]	1.61	1.28	[−0.91, 4.12]
Gender	0.70	5.49	[−10.06, 11.47]	1.06	5.39	[−9.51, 11.63]
Age	−0.39^**^	0.15	[−0.68, −0.10]	−0.35^*^	0.15	[−0.64, −0.06]
Teaching experience	0.05	0.16	[−0.25, 0.36]	0.10	0.15	[−0.20, 0.40]
Title	−0.85^*^	0.37	[−1.59, −0.12]	−1.00^**^	0.37	[−1.72, −0.28]
Empathic ability	—	—	—	−0.60^***^	0.15	[−0.90, −0.30]
Empathic motivation	—	—	—	−0.90^***^	0.25	[−1.40, −0.41]
	*R*^2^ = 0.018, △*R*^2^ = 0.018, *F* = 4.2^***^			*R*^2^ = 0.055, △*R*^2^ = 0.038, *F* = 22.89^***^		

^*^p < 0.05, ^**^p < 0.01, ^***^p < 0.001. The regression coefficients are non-standardized.

### 3.3 Latent path model

**Figure 1:**
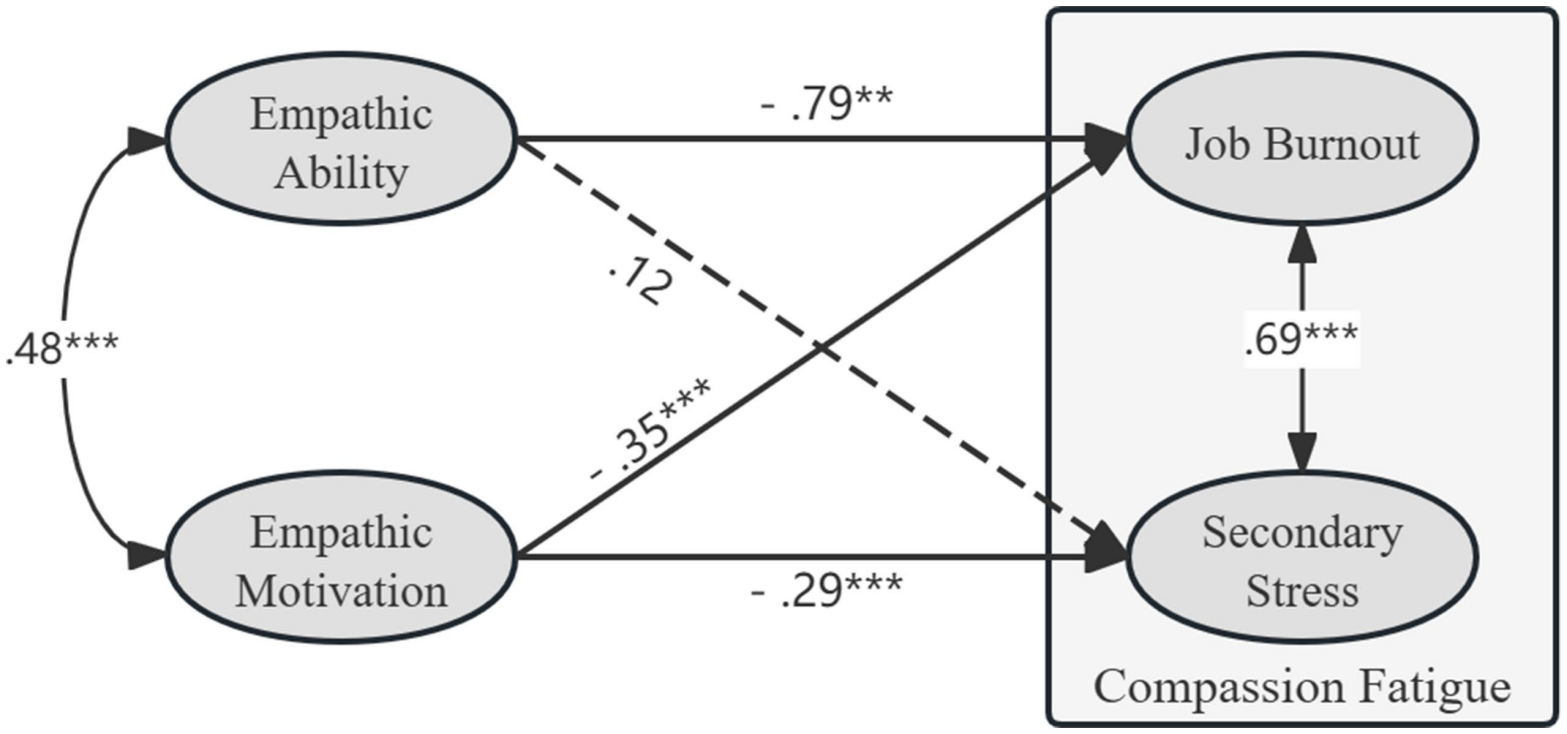
The multivariate multiple regression model is among the major variables. The regression coefficients are non-standardized. The solid lines denote significant coefficients, and the dashed line denotes insignificant effects. ^**^p < 0.01, ^***^p < 0.001.

As shown in Table 3 and Figure 1, to further test the predicted effects of empathic ability and empathic motivation on the subscales of compassion fatigue, a latent path model in a structural equation (SEM) framework was constructed after controlling for the covariates. The confirmatory factor analysis (CFA) indicated that all factor loadings for the compassion fatigue scale ranged from 0.51 to 0.87 and were statistically significant (*p* < 0.001), supporting the construct validity of this scale. The analysis showed that the model fit was *χ*^2^/*df* = 5.33, RMSEA = 0.061, 95% CI [0.058, 0.064], CFI = 0.88, TLI = 0.87, and SRMR = 0.058. Although the RMSEA indicated acceptable fit, the CFI and TLI were slightly below the recommended cutoff values, suggesting a limited acceptable model fit (Hu and Bentler, 1999). Furthermore, job burnout was significantly and negatively predicted by empathic ability (*β* = −0.79, *SE* = 0.28, *p* < 0.01, 95% CI [−1.36, −0.26]) and empathic motivation (*β* = −0.35, SE = 0.9, *p* < 0.001, 95% CI [−0.53, −0.19]). Secondary stress was significantly and negatively predicted by empathic motivation (*β* = −0.29, *SE* = 0.08, *p* < 0.001, 95% CI [−0.45, −0.12]); however, the predictive relationship between secondary stress and empathic ability was not significant (*β* = 0.12, *SE* = 0.29, *p* = 0.67, 95% CI [−0.40, 0.73]). The predictors in the SEM collectively accounted for 8.9% of the variance in job burnout and 2.6% of the variance in secondary stress. These values indicate that the model captures a modest but meaningful proportion of variability in the outcome variables. In addition, the results also indicated that age and title significantly predicted job burnout, with older teachers reporting lower burnout (*β* = −0.05, *p* < 0.001) and higher title associated with lower job burnout (*β* = −0.09, *p* = 0.009). In contrast, areas and gender were not significant predictors of burnout (*β* = 0.102, *p* = 0.38; *β* = 0.26, *p* = 0.58, respectively), and teaching experience was also non-significant (*β* = 0.010, *p* = 0.388). Meanwhile, for secondary stress, none of the demographic variables showed significant predictive effects. Areas (*β* = 0.14, *p* = 0.196), gender (*β* = −0.14, *p* = 0.736), age (*β* = 0.01, *p* = 0.686), teaching experience (*β* = 0.008, *p* = 0.524), and title (*β* = −0.05, *p* = 0.106) were all non-significant. These findings suggest that demographic factors had a limited impact on secondary stress in this sample, whereas age and professional title were relevant for job burnout.

**Table 3:** Testing the pathways of latent path model.

Pathways	*β*	SE	95% Boot LLCI	95% Boot ULCI
Empathic ability → Job burnout	−0.79^**^	0.28	−1.36	−0.26
Empathic motivation → Job burnout	−0.35^***^	0.09	−0.53	−0.19
Empathic ability →Secondary stress	0.12	0.29	−0.40	0.73
Empathic motivation → Secondary stress	−0.29^***^	0.08	−0.45	−0.12

^**^p < 0.01, ^***^p < 0.001. 95% Boot LLCI and 95% Boot ULCI represent the lower and upper limits, respectively, of the 95% bias-corrected bootstrap confidence interval for each estimate.

## 4 Discussion

This study aimed to resolve the question of whether compassion fatigue is driven by a deficit in empathic ability, motivation, or both. Accordingly, a cross-sectional study was conducted, and the hypotheses were examined using structural equation modeling for the purpose of data analysis and hypothesis testing. The results showed that compassion fatigue was significantly negatively predicted by empathic ability and empathic motivation. Specifically, regarding the two dimensions of compassion fatigue, job burnout was significantly and negatively predicted by both empathic ability and empathic motivation, whereas secondary trauma was significantly and negatively predicted by empathic motivation but not by empathic ability.

These findings indicate that increased compassion fatigue is accompanied by a decrease in empathic ability and empathic motivation. This empirically corroborates the theoretical foundations laid by Figley (1995) and Adams et al. (2006), who conceptualized compassion fatigue as a caregiver’s diminished capacity or willingness to engage empathically with clients’ suffering. This perspective is further aligned with Sun et al. (2011), who described it as a decline in both the interest and the ability to empathize. Building upon this evidence, the present study offers several key strengths.

Theoretically, empathy deficit is a key feature of compassion fatigue. Our finding that compassion fatigue decreases with higher empathic ability and motivation provides direct empirical support for this view. This result echoes the empathic motivational framework (Zaki, 2014) and empathic propensity-ability dissociating theory (Keysers and Gazzola, 2014), which holds that empathy is often a motivational phenomenon, and empathy deficit is the result of not only the absence of empathic ability but also empathic motivation. The results of this study provide direct evidence to support these theories. Furthermore, the findings confirm that empathy is a significant component of teachers’ professional identity (Peck et al., 2015; Stojiljković et al., 2012). As an outcome of empathic satisfaction, a decline in empathy suggests a weakened professional identity and a greater likelihood of compassion fatigue. However, we also found that secondary stress, a subcomponent of compassion fatigue, was not predicted by empathic ability. This may be explained by the nature of secondary stress, which is characterized by symptoms of fear, intrusive thoughts, and avoidance resulting from indirect exposure to others’ trauma (Figley, 2002; Ormiston et al., 2022). Unlike the more immediate emotional resonance associated with empathic ability, secondary stress emphasizes profound shifts in cognitive schemas, such as changes in beliefs about oneself, others, and the world (Pearlman and Mac Ian, 1995; White, 2006). Thus, secondary stress may be more closely linked to motivational and belief-based factors (e.g., empathic motivation) than to the capacity for empathy itself. Nonetheless, the relationships between the secondary stress and empathic motivation are far from settled and represent an area for future research.

Despite its strengths, this study has several limitations. First, this study adopted only self-reported assessments, which might be susceptible to response bias, such as social desirability. Further research is needed to collect more objective indicators of major variables related to compassion fatigue in kindergarten teachers. Second, this study’s sample was limited to the kindergarten teacher group. Although kindergarten teachers are also vulnerable to empathy fatigue, whether these research results can be extended to other groups, such as surgeons, nurses, and police officers, requires more in-depth research in the future. Finally, the gender ratio was not balanced in this sample because, in fact, there are more women than men in preschool education in China. Future research should focus on the problem of CF among male kindergarten teachers. Importantly, compassion fatigue arises from the interaction of multiple factors. Future studies should also focus on influences like workplace support, child-to-teacher ratios, and personal trauma.

## 5 Conclusions and recommendations

This study establishes that compassion fatigue is jointly influenced by deficits in both empathic ability and motivation, with empathic motivation demonstrating a broader impact across its dimensions. While job burnout is associated with limitations in both ability and motivation, secondary trauma appears predominantly linked to motivational factors. These findings clarify long-standing theoretical questions regarding the empathic underpinnings of compassion fatigue and highlight the need for comprehensive intervention strategies that address both components. Future research should further explore the mechanisms underlying these distinct pathways and develop targeted approaches to mitigate the “cost of caring” among caregiving professionals.

This study has some practical implications as well. First, our findings support prior research identifying teachers as a population vulnerable to compassion fatigue, thereby underscoring the practical significance of this line of inquiry. Given that compassion fatigue poses a serious threat to mental and physical health, the question of how to alleviate it remains pressing. Our results suggest that interventions should target not only empathic ability but also empathic motivation. In this vein, researchers have begun exploring motivational approaches to address empathy deficits, such as mindset-based (Schumann et al., 2014), norm-based (Weisz et al., 2021), and reward-based interventions (Ferguson et al., 2020). Among these, interventions targeting malleable empathy mindsets offer a promising approach to addressing compassion fatigue. For example, empirical evidence confirms that individuals who view empathy as a developable capacity demonstrate stronger empathic motivation than those with fixed mindset (Schumann et al., 2014). Systematic interventions cultivating this malleable mindset have effectively enhanced empathic motivation and observable behaviors across diverse populations (Weisz et al., 2022; Weisz et al., 2021). Thus, these findings suggest that cognitive restructuring of empathy beliefs may serve as a viable pathway for boosting empathic motivation and potentially mitigating compassion fatigue.

## Data Availability

The raw data supporting the conclusions of this article will be made available by the authors, without undue reservation.

## Footnotes

1
https://www.wjx.cn/
